# Human Cytomegalovirus Induces Vitamin-D Resistance In Vitro by Dysregulating the Transcriptional Repressor Snail

**DOI:** 10.3390/v14092004

**Published:** 2022-09-10

**Authors:** Carmen Stecher, Katharina Philomena Maurer, Marie-Theres Kastner, Christoph Steininger

**Affiliations:** 1 Department of Medicine I, Division of Infectious Diseases and Tropical Medicine, Medical University of Vienna, 1090 Vienna, Austria; 2 Karl-Landsteiner Society, Institute of Microbiome Research, 3100 St. Pölten, Austria

**Keywords:** VDR, HCMV, cytomegalovirus, vitamin D, calcitriol, Snail, cathelicidin, LL-37

## Abstract

Vitamin-D supplementation is considered to play a beneficial role against multiple viruses due to its immune-regulating and direct antimicrobial effects. In contrast, the human cytomegalovirus (HCMV) has shown to be resistant to treatment with vitamin D in vitro by downregulation of the vitamin-D receptor. In this study, we aimed to elucidate the mechanism and possible biological consequences of vitamin-D resistance during HCMV infection. Mechanistically, HCMV induced vitamin-D resistance by downregulating the vitamin-D receptor (VDR) within hours of lytic infection. We found that the VDR was inhibited at the promoter level, and treatment with histone deacetylase inhibitors could restore VDR expression. VDR downregulation highly correlated with the upregulation of the transcriptional repressor Snail1, a mechanism likely contributing to the epigenetic inactivation of the VDR promoter, since siRNA-mediated knockdown of Snail partly restored levels of VDR expression. Finally, we found that direct addition of the vitamin-D-inducible antimicrobial peptide LL-37 strongly and significantly reduced viral titers in infected fibroblasts, highlighting VDR biological relevance and the potential of vitamin-D-inducible peptides for the antiviral treatment of vitamin-D deficient patients.

## 1. Introduction

In addition to its classic role in calcium and phosphorus homeostasis, vitamin D3 has been increasingly acknowledged for its role in immunity and modulation of antiviral defenses. Specifically, it inhibits antigen-mediated T-cell activation [1], can trigger antiretroviral autophagy in macrophages [2] and potently induces antimicrobial peptides such as beta-defensin and cathelicidin in neutrophils and other immune cells [3]. Vitamin D exerts the majority of its effects via the Vitamin-D receptor (VDR), a nuclear hormone receptor which forms a heterodimeric transcription factor with the Retinoid-X receptor alpha (RXRα) to act on promoters that contain vitamin-D response elements. Binding of calcitriol, the bioactive form of vitamin D, further enhances VDR transcriptional activity [4]. The activated VDR can mediate direct antimicrobial effects, for example, by its induction of the antimicrobial peptide cathelicidin (also LL-37 or hCAP-18), which has been shown to inhibit Pseudomonas aeruginosa, Staphylococcus aureus, Adenovirus and Herpes simplex virus 1 in vitro [5]. Vitamin-D deficiency has also been discussed as a factor of disease severity in the context of human cytomegalovirus infection, which poses a serious problem, especially in organ transplantation [6]. Graft rejection by itself is associated with an insufficient cholecalciferol nutritional status, and in-vivo data from animal experiments suggest that vitamin-D supplementation may decrease acute allograft rejection after solid organ transplantation [7,8,9]. In patients, there is evidence that supplementation with calcitriol, the bioactive form of vitamin D, leads to amelioration of acute cellular rejection rates and may simplify treatment options in kidney transplant patients [10,11,12]. However, we have recently found that the human cytomegalovirus downregulates the vitamin-D receptor (VDR) during infection [13], thus conferring cellular resistance to bioactive vitamin D. Additionally, VDR levels were found to be generally reduced in the blood cells of hematopoietic stem-cell transplant patients undergoing active HCMV infection [14]. Mechanistically, it is not known how HCMV influences VDR expression, or whether this might benefit viral propagation. Here, we investigated how the VDR is repressed during HCMV infection, identified potential implications of epigenetic VDR repressors, and discuss potential biological implications of the repression of vitamin D in HCMV-infected cells.

## 2. Materials and Methods

### 2.1. Tissue Culture

Primary human foreskin fibroblasts (abbreviated as HFF) and ARPE-19 retinal pigment epithelial cells (ATCC CRL-2302) were cultured at 37 °C, 5% CO_2_ in Dulbecco’s modified eagle medium (DMEM GlutaMAX, Gibco, Thermofisher Scientific, Waltham, MA, USA) supplemented with 10% fetal bovine serum (FBS, Gibco) and a penicillin/streptomycin mix (Gibco, 100 U/mL). HFF were propagated between passages 8–20 to avoid replicative senescence. ARPE-19 were subcultured every 2–3 days at a ratio of 1:5.

### 2.2. HCMV Infection

Human cytomegalovirus (HCMV, strain AD169) was prepared as described previously [15]. For virus production and measurement of viral genomic DNA, HFF were grown to a confluent monolayer and infected with HCMV at a multiplicity of infection (MOI) of 0.01 to 0.05. After 1.5 h, the viral inoculum was replaced by fresh growth medium. The viral supernatant was harvested upon visibility of a total cytopathic effect, usually 8–10 days post infection, and stored at −80 xB0;C. After thawing, cellular material was removed by centrifugation at 3000× *g* for 20 min. Infectivity of the viral inoculum was then measured using a plaque assay [16]. HCMV strain TS15-rN (kindly provided by Sallie R. Permar) was used to infect ARPE-19 cells.

For infection of cell cultures with HCMV AD169, cells were seeded into 6-well plates overnight, and inoculated at MOI 1 (if not indicated otherwise) for 1 h at 37 °C. After viral adsorption, the cell layer was rinsed and fresh growth medium including 10% FBS was added to the cells. In LL-37 treatment experiments (see below), growth medium containing 2% FBS was resupplied after inoculation. For ganciclovir-treated control wells, Ganciclovir (Merck Millipore, Burlington, MA, USA) was added to the growth media at a concentration of 10 μg/mL after removal of the inoculum.

### 2.3. UV Inactivation

For UV inactivation of virus, pre-titered CMV AD169 supernatant was pipetted into the center of an ice-cooled petri dish. The uncovered suspension was irradiated in a Bio-Link BLX UV crosslinker for 45 min at a distance of approximately 20 cm. Virus inactivation was validated by the absence of transcription of immediate early genes (IE1, IE2) by quantitative PCR twelve hours post infection, and/or by validating the absence of viral IE72 protein using Western blot.

### 2.4. RNA Isolation and RT-qPCR

RNA was extracted from cells using the RNeasy kit from Qiagen according to the manufacturer’s instructions, including the on-column DNA digest using RNAse free DNase (Qiagen, Hilden, Germany). First strand cDNA was synthesized using iScript reverse transcriptase (Biorad, Hercules, CA, USA) and the accompanying buffer, which includes a mixture of random hexamer and oligo-dT primers.

Quantitative reverse transcription PCR (qRT-PCR) was performed using a StepOne Plus Real-Time PCR System (Applied Biosystems, Waltham, MA, USA) and Applied Biosystems’ Power SYBR Green Master Mix. Sequence-specific oligonucleotide primers were synthesized by Microsynth (Austria). Relative expression values were normalized to human GAPDH, SDHA and PGK1, using the comparative threshold cycle method (2^−ΔCt^ or 2^−ΔΔ^Ct) for comparison of mRNA expression between samples (e.g., infected versus mock-infected). Primers used are described in Table S1.

### 2.5. SDS-PAGE and Western Blot

For the production of whole cell protein lysate, cell layers were washed with PBS and lysed with 50 mM Tris pH 7.5, 500 mM NaCl, 1% NP-40, 0.1% SDS containing buffer supplemented with 1% of a Protease and Phosphatase Inhibitor (Halt, Thermofisher Scientific ). Lysates were sonicated for 4 pulses of 10 s in a Bioruptor Plus (Diagenode, Denville, NJ, USA) bath sonicator and clarified by centrifugation at 10,000× *g* for 7 min. Subsequently, protein concentration of lysates was determined using the Pierce BCA protein assay kit (Thermofisher Scientific). Equal amounts (between 10 and 30 μg) of sample were loaded onto self-cast 6%/8% polyacrylamide gels or 4–15% precast Mini Protean TGX polyacrylamide gradient gels (Biorad) and subjected to SDS-PAGE.

For phosphorylation-specific separation of Snail1 protein, we performed Phos-Tag SDS-PAGE using pre-cast Wako SuperSep Phos-Tag 7.5% (50 μM) gels (Fujifilm, Tokyo, Japan). Gels were run at constant current of 15 mA until the loading front ran out. Before blotting, gels were washed for 6 × 10 min in 10 mM EDTA-Tris/Glycine buffer and equilibrated for 2 × 10 min in 1 mM EDTA Tris/Glycine buffer. Proteins were then blotted to a 0.45 μm PVDF membrane (Thermofisher Scientific) in a wet tank (Tris/Glycine buffer containing 20% methanol) for 1 h at 100 V. Phos-Tag gels were blotted in Tris/Glycine transfer buffer containing 5% *v/v* methanol and 1 mM EDTA overnight at 24 V. Membranes were then stained with Ponceau S solution for verification of blotting efficiency and visualization of total protein amount. Destained membranes were blocked in StartingBlock TBS blocking buffer (Thermofisher Scientific) and probed with primary antibody overnight at 4 °C. After HRP-linked secondary antibody incubation and washes in 1× TBS, blots were imaged on a ChemiDoc (Biorad) using the SuperSignal Pico or Femto substrates from Thermofisher Scientific.

The monoclonal antibodies RXRα #3085, Snail #3879, Slug #9585, VDR #12550, polyclonal antibody against DNA-PKcs #4602 and secondary goat α-Rabbit HRP #7074 were obtained from Cell Signaling Technology. The monoclonal antibodies GAPDH sc-47724, Ubiquitin sc-8017, HCMV pp65 sc-56976, pp72/86 sc-69748, pp86 sc-69835 were from Santa Cruz Biotechnologies. Secondary goat anti-mouse HRP antibody was from Biorad (1706516). The UL32/pp150 IgG2b monoclonal antibody was synthesized from hybridoma cell lines and stems from mice immunized with the XP1 antigen expressed in E. coli, as described previously [17].

### 2.6. Plasmid Cloning and Transfection

The ORF of IE2 was first amplified from cDNA of infected HFF (24 h p.i.) using an iProof polymerase kit (Biorad). Restriction sites (BamHI/XbaI containing primers) were added in a second PCR step to the resulting amplicon for subsequent insertion into the pcDNA3.1/Hygro(+) vector (Thermofisher Scientific). Sequence identity of the insert with the AD169 IE2 CDS (NCBI FJ527563.1) was confirmed by Sanger sequencing of both the forward and reverse strand.

HEK293 cells were transfected with 850 ng of pcDNA-IE2 or empty vector control plasmid in 24-well plates using 2 μL Turbofect (Thermofisher Scientific) according to the manufacturer’s instructions. Two days post transfection, lysates were harvested for analysis via Western blot.

### 2.7. siRNA Transfections

Single IE1/2, DNA-PKcs and Snail1-targeting siRNAs as well as non-targeting control siRNA siCTRL were produced by Microsynth (Austria) with 3’ dTdT overhangs (see Table S2). Snail1 siRNA mix sc-38398 (containing 3–5 different siRNAs targeting the same mRNA) was obtained from Santa Cruz Biotechnology. For transfections, HFF were seeded into 6-well plates and single or double transfected at ≥80% confluence with a mixture of 50 nM siRNA and 5 μL Turbofect (Thermofisher Scientific) diluted in serum-free and antibiotic-free Optimem medium (Thermofisher Scientific). Five–six h after transfection, cells were washed and the medium was replaced with fresh growth medium. Cells were transfected a second time after 24 h adhering to the same protocol. Another 24 h later, HFF were infected with HCMV AD169 at an MOI of 1 and harvested at the indicated time points for analysis of protein expression.

### 2.8. Cathelicidin Treatment

Cathelicidin (LL-37/hCAP-18) peptide was obtained from Invivogen (tlrl-l37). Treatment of virus or pre-treatment of cells was performed in serum-free medium at the indicated concentrations. Growth medium containing 2% FBS was resupplied after virus inoculation.

### 2.9. Alamar Blue Viability Assay

AlamarBlue Cell Viability Reagent (Thermofisher Scientific)was used according to the manufacturer’s instruction to determine relative viability of LL-37-treated cells. To achieve this, cells were seeded as usual, treated with the indicated concentrations of LL-37 peptide in the absence of serum and infected with CMV AD169 (MOI = 1). At 16 h p.i., the medium was replaced by full growth medium containing the AlamarBlue Cell Viability Reagent at a 1 to 10 dilution. After 4 h incubation at 37 °C, fluorescence was measured on a Varioskan plate reader (Thermofisher Scientific)at an excitation maximum of 560 nm and emission maximum of 590 nm for 10 ms. Fluorescence values were normalized to the values of untreated mock- and CMV-infected cells, respectively.

### 2.10. Functional Plaque Reduction Assays

For the functional plaque reduction assays using cathelicidin treatment, HFF were seeded into 24-well plates and pre-treated with vehicle (ddH2O) or LL-37 (Invivogen) for 1 h in serum-free growth medium at the indicated concentrations. The cells were then incubated with viral inoculum at 60 plaque forming units per well for 90 min. Subsequently, the cells were overlaid with growth medium containing 0.5% low-melting-point agarose. After solidification of the agarose layer, the plates were incubated for 10 days at 37 °C in a CO_2_ incubator, then fixed with 2% formaldehyde, stained with 0.02% methylene blue and analyzed for number of plaques under a light microscope. Plaque numbers were normalized to the mean of vehicle-treated control samples.

For plaque reduction assays using Snail-targeting siRNAs, cells seeded into 24-well plates the day before were transfected with two different siRNA targeting Snail1 or a non-targeting control siRNA siCTRL, as described above. After an overnight incubation, plates were then infected with 60 pfu per well and overlaid with low-melting agarose as previously described.

### 2.11. Chromatin Immunoprecipitation

Chromatin immunoprecipitation from mock- and CMV-infected cells was performed using the SimpleChIP Enzymatic Chromatin IP Kit (Magnetic Beads) from Cell Signaling Technology according to the manufacturer’s protocol. A minimum of 4 × 106 cells were prepared per IP. Chromatin was enzymatically digested using micrococcal nuclease. Additionally, nuclear lysis was ensured by sonciation pulses of 30 s in a Bioruptor Plus bath sonicator (Diagenode) and verified by analysis of an aliquot of the lysate under a light microscope. A total of 10 to 15 μg chromatin diluted in StartingBlock TBS blocking buffer (Thermofisher Scientific) were used per IP. Furthermore, 2 μg of H3K27-specific ab4729 (Abcam, Cambridge, UK) or rabbit IgG control antibody (Cell Signaling, Danvers, MA, USA) was incubated overnight at 4 °C with gentle rotation. Chromatin fragment quality was routinely analyzed by reverse crosslinking of an aliquot and separation via gel electrophoresis.

Quantitative PCR of ChIP and 2% input control samples (ChIP-qPCR) was performed using a StepOne Plus Real-Time PCR System using PowerUp SYBR Master Mix (Applied Biosystems) according to the manufacturer’s instructions. VDR promoter enrichment was calculated relative to adjusted (100%) input.

For conventional PCR, iTaq polymerase kit (Biorad) was used for VDR promoter amplification (30 cycles at 98 °C 30 s, 59 °C 30 s, 72 °C 30 s). Amplicons were then separated on a 1% agarose gel including GelRed (Biotium, Fremont, CA, USA) in TAE buffer and imaged on a ChemiDoc (Biorad).

### 2.12. Statistical Analyses

All bar graphs and error bars show the mean ± SEM of 3 or more independent experiments, if not explicitly indicated otherwise. The IC50 and 95% confidence intervals for inhibition curves were calculated with Graphpad Prism 7.00 to fit a variable slope sigmoidal dose–response curve. Data were analyzed using Graphpad Prism 7.00 using the respective statistical analysis and post-hoc test indicated in the figure legends. *p* values smaller than 0.05 were considered significant (* = *p* < 0.05, ** = *p* < 0.01, *** = *p* < 0.001, **** = *p* < 0.0001).

## 3. Results

### 3.1. The VDR Is Epigenetically Repressed during HCMV Infection

**Figure 1:**
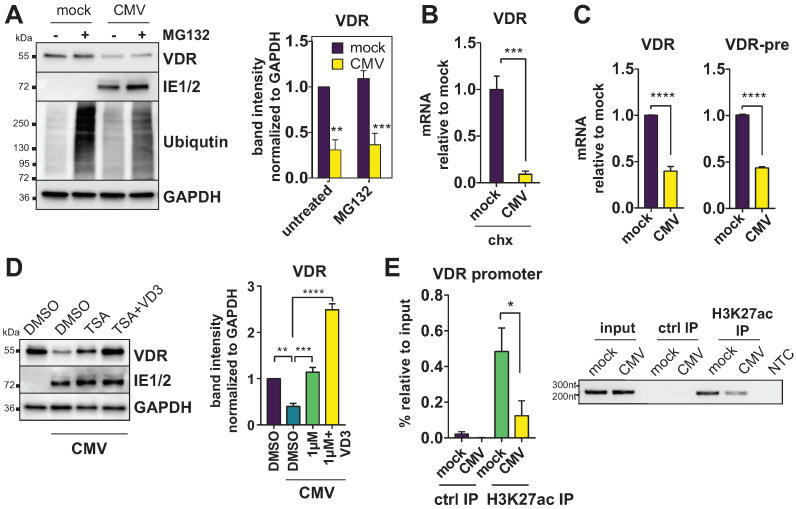
The VDR is epigenetically repressed during HCMV infection. (**A**) Immunoblot showing VDR and GAPDH expression in HFF treated with DMSO (−) or 1 μM MG132 (+) proteasome inhibitor in mock- or CMV-infected cells. Ubiquitinated proteins are shown as a control for proteasome inhibitor effectiveness. Representative blot (left) and bar graph from multiple experiments (right panel). (**B**) RT-qPCR analysis of VDR mRNA expression in mock- or CMV-infected HFF treated with 50 μg/mL cycloheximide (chx, n = 2) starting at 6 h p.i. and harvested 24 h p.i. Asterisks indicate significant differences to the mock control sample (unpaired Student’s *t* test). (**C**) RT-qPCR analysis of VDR and intron-containing unspliced pre-mRNA (VDR-pre) expression in mock- and HCMV-infected HFF harvested 24 h p.i. Asterisks indicate significant differences to the mock control sample using unpaired Student’s *t* tests. (**D**) Immunoblots showing VDR expression compared to GAPDH loading control in HFF infected with CMV and treated with DMSO (vehicle), 1 μM TSA, or a combination of TSA and 10 nM calcitriol (VD3), harvested 24 h p.i. A representative blot is shown on the left, the right panel shows VDR quantification from multiple experiments with significant differences to the CMV DMSO control sample as determined by ANOVA Dunnett’s multiple comparison post-hoc test. (**E**) Purified chromatin from mock- and CMV-infected HFF was immunoprecipitated with an anti-H3K27ac polyclonal antibody or normal goat serum (ctrl IP). The left panel shows qPCR results from three independent experiments. On the right, VDR promoter amplicons from a Taq-PCR were separated on an agarose gel. NTC = no template control.

Recently, we have shown that HCMV infection downregulates the VDR and that calcitriol and other forms of vitamin D are not able to directly inhibit HCMV AD169 propagation in infected fibroblasts [13]. To further our understanding of the mechanism of this HCMV-induced VDR downregulation, we, thus, first determined at what step of synthesis VDR inhibition takes effect. First, we treated infected human-foreskin fibroblasts (HFF) with the proteasome inhibitor MG132 to assess whether protein degradation plays a role in the loss of VDR. However, MG132 treatment could not rescue VDR reduction (Figure 1A), excluding proteasomal degradation as a main effector of downregulation. Similarly, we aimed to exclude an effect of increased VDR mRNA stability during HCMV infection. When treated with cycloheximide, a protein synthesis inhibitor known to prevent the degradation of labile mRNAs, we could still observe significantly less VDR mRNA in infected cells (Figure 1B). In fact, we did not only measure decreased levels of VDR mRNA in infected cells, but similarly downregulated unspliced pre-mRNA levels (Figure 1C), indicating that the VDR might be inhibited at the mRNA transcription step. Finally, we determined that treatment of HCMV-infected HFF with the histone deacetylase (HDAC) inhibitor trichostatin A (TSA) could rescue VDR protein levels to normal levels (Figure 1D), suggesting that the VDR is likely inhibited at the promoter level via an epigenetic silencing mechanism. A chromatin immunoprecipitation (ChIP) assay using H3K27-acetylation-specific antibodies against chromatin from mock- and HCMV-infected HFF suggested that the VDR promoter is significantly less acetylated at Lysine 27 of histone H3 in HCMV infected cells, further supporting the notion of VDR epigenetic repression (Figure 1E).

### 3.2. HCMV Regulates the VDR Repressors Snail1 and Snail2

**Figure 2:**
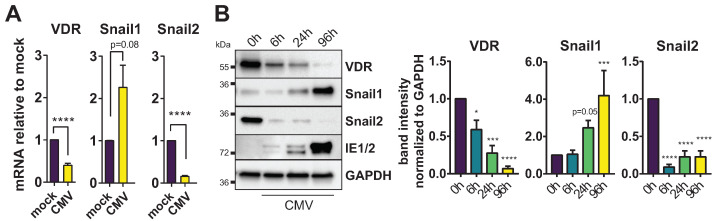
HCMV regulates the VDR repressors Snail1 and Snail2. (**A**) RT-qPCR analysis of VDR, Snail1 and Snail2 mRNA expression in mock- and CMV-infected HFF is shown. Asterisks indicate significant differences to the mock control sample (unpaired Student’s *t* test). (**B**) Immunoblots showing VDR, Snail1, Snail2 and IE1/2 expression compared to GAPDH loading control in HFF infected with CMV and harvested at the indicated time point p.i. Representative blot and quantification from multiple experiments with significant differences to the mock (0 h) control sample as determined by ANOVA Dunnett’s multiple comparison post-hoc tests.

While the VDR is known to regulate more than 200 target genes [18], less is known about the regulation of the VDR promoter itself. The epithelial-to-mesenchymal (EMT) transcription factors Snail1 and Snail2 (or Snail and Slug) have been shown to directly repress the VDR promoter in the context of cancer [19,20]. Thus, we next tested the induction of Snail1 and Snail2 in the context of HCMV infection. It was previously reported that EMT markers are downregulated by HCMV [21]. Indeed, we could observe a strong reduction in Snail2 mRNA and protein during infection (Figure 2A,B). However, expression of its homolog Snail1 inversely correlated with Snail2 and VDR expression, as it was strongly upregulated during infection, especially on the protein level (Figure 2B). In addition to its role as a cancer EMT marker, Snail influences various cellular differentiation processes, e.g., cell-fate decisions during embryogenesis and wound healing or the repression of tight junctions [22,23,24], processes potentially relevant during viral infections.

### 3.3. VDR Holoenzyme Downregulation Is Dependent on Immediate Early Gene Transcription

**Figure 3:**
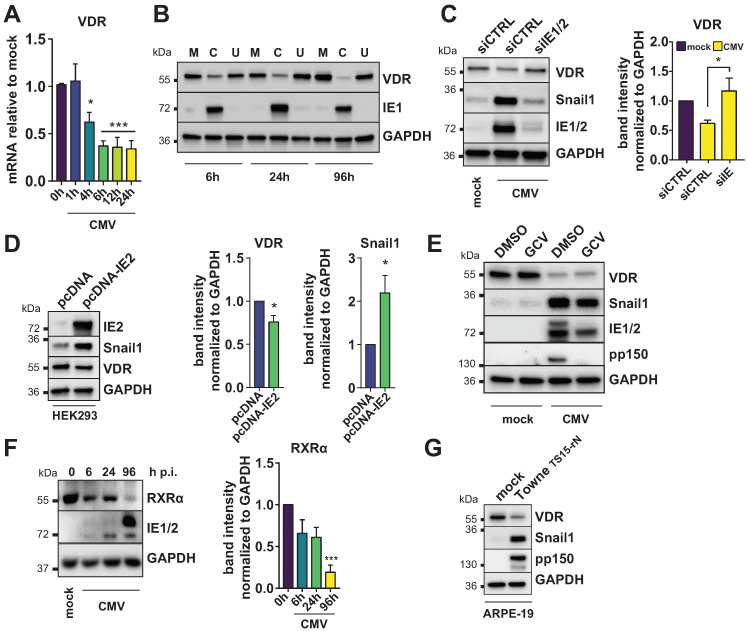
VDR holoenzyme downregulation is dependent on immediate early gene transcription. (**A**) Vitamin-D-receptor expression was measured in mock- or HCMV-infected HFF at the indicated time points, showing fold change (2^−ΔΔ^Ct) compared to mock-infected cells (mean ± SEM). Asterisks indicate significant differences to the mock control sample as determined by ANOVA Dunnett’s multiple comparison post-hoc test. (**B**) VDR, IE1 (pp72) and GAPDH protein expression were determined by Western blot in HFF that were infected with mock (M), CMV (C) and UV-inactivated CMV (U) inocula and harvested at the indicated times post infection. (**C**) VDR and HCMV immediate early protein expression was determined in lysates of HFF transfected with a non-targeting control siRNA or IE1/2 specific siRNA harvested at 24 h p.i. Representative blot (left) and quantification of bands from multiple experiments relative to the GAPDH loading control (right). Asterisks indicate significant differences to the CMV siCTRL sample as determined by ANOVA Dunnett’s multiple comparison post-hoc test. (**D**) HEK293 cells were transfected with pcDNA empty vector control or pcDNA containing the IE2 open reading frame. Lysates were analyzed by Western blot at 48 h post transfection. Asterisks indicate significant differences to empty vector control (unpaired t-test). (**E**) VDR and Snail1 expression was determined at 96 h p.i. in lysates of HFF treated with vehicle (DMSO) or 10 μg/mL Ganciclovir (GCV). HCMV immediate early and pp150 late antigens were stained as a control. (**F**) RXRα protein expression in HFF was determined by specific immunoblots at the indicated time points p.i. Representative blot (left) and quantification of bands from multiple experiments relative to the GAPDH loading control (right, one-way ANOVA and Dunnett’s post-hoc test). (**G**) ARPE-19 cells were infected with mock supernatant or the pentamer-positive (TS15-rN) variant of the Towne strain and harvested 14 d p.i. VDR, Snail1, pp150 late antigen and a GAPDH loading control detection by Western blot is shown.

In order to narrow down on the molecular mechanism behind HCMV-mediated VDR downregulation, we next analyzed the temporal dynamics of VDR and Snail1 expression compared to the viral transcriptional cascade in HFF infected with the laboratory HCMV strain AD169. Using RT-qPCR, we observed that VDR mRNA was significantly downregulated as early as 4 h post infection (Figure 3A). Additionally, viral transcription seemed to be necessary for VDR downregulation, since the infection with UV-inactivated HCMV incapable of immediate early transcription did not lead to reduced VDR levels at different time points throughout infection, as determined by Western blot (Figure 3B). Similarly, siRNA-mediated knockdown of the HCMV immediate early genes IE1 and IE2 attenuated VDR protein reduction in infected fibroblasts (Figure 3C). To elucidate whether immediate early expression was not only necessary, but also sufficient for VDR repression, we cloned the IE2 open reading frame into an overexpression plasmid and transfected HEK293 cells. Indeed, overexpression of IE2 led to a significant increase in Snail1 and a, albeit small, reduction in VDR protein levels (Figure 3D). Importantly, treatment with the nucleoside analog Gancliclovir (GCV)—which exerts its antiviral effects after the immediate early phase at the DNA replication step—could not rescue VDR downregulation or Snail1 upregulation (Figure 3E).

Since the VDR employs its functions mostly as a heterodimeric holoenzyme with RXRα, we also analyzed RXRα expression in the context of HCMV infection. RXRα protein showed to be similarly reduced by HCMV, especially at late time points of infection (Figure 3F), indicating that loss of RXRα might also contribute to CMV-induced vitamin-D resistance.

Of note is that Snail upregulation in combination with VDR reduction was also observed when infecting retinal pigment epithelial (ARPE-19) cells with an epithelial-permissive strain of CMV Towne (Figure 3G), indicating that these changes are likely not entirely strain- or cell-type restricted.

### 3.4. Snail Is Stabilized by DNA-PK and Snail Ablation Increases VDR Expression

**Figure 4:**
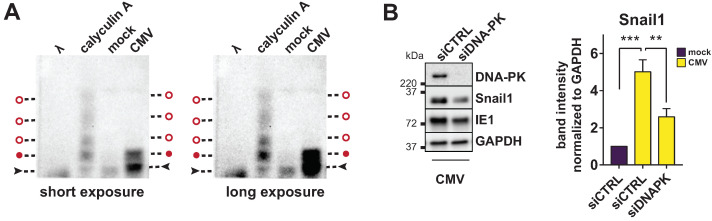
Snail phosphorylation and regulation by DNA-Protein kinase. (**A**) Lysates from mock- and HCMV-infected cells were harvested at 24 h p.i. and subjected to Phos-Tag SDS-PAGE. As additional controls, CMV-infected HFF were treated with a phosphatase inhibitor for 2 h before lysis (calyculin A), or with lambda phosphatase for 30 min λ). A representative Snail1-specific immunoblot shows the ratio between unphosphorylated (black arrows) and phosphorylated Snail1 (red circles). The same blot is shown with short (left) and long exposure (right). Height of phosphorylated bands also present in the CMV condition is marked with full red circles, open red circles indicate further (weak) phosphorylation bands in the calyculin A treatment control. (**B**) Representative Western blot showing DNA-PK and Snail protein expression determined in lysates of HCMV-infected HFF transfected with a non-targeting control siRNA or DNA-PK specific siRNAs and harvested at 24 h p.i. (representative Western blot on the left and quantification relative Snail1 band intensity of multiple independent experiments on the right; asterisks show significant differences to the CMV siCTRL sample as determined by ANOVA Dunnett’s multiple comparison post-hoc test).

Snail1 is an inherently unstable protein heavily regulated by post-translational modifications by targeted degradation, with a half-life of only about thirty minutes under steady-state conditions [25,26]. Indeed, we found that Snail1 was partly phosphorylated in CMV-infected HFF lysates (Figure 4A) by separation on a Phos-Tag SDS-PAGE gel, indicating that it might be post-translationally regulated during infection. One of the few kinases known to have a stabilizing effect on Snail1 is the catalytic subunit of the DNA-dependent Protein Kinase (DNA-PK) [27]. Therefore, we next tested whether Snail1 was still induced under conditions in which DNA-PK was knocked down. Indeed, Snail1 protein abundance was reduced in HCMV-infected HFF when DNA-PK was knocked down by specific siRNAs compared to a non-targeting control (Figure 4B), indicating that DNA-PK might play a role in Snail1 stabilization during HCMV infection.

**Figure 5:**
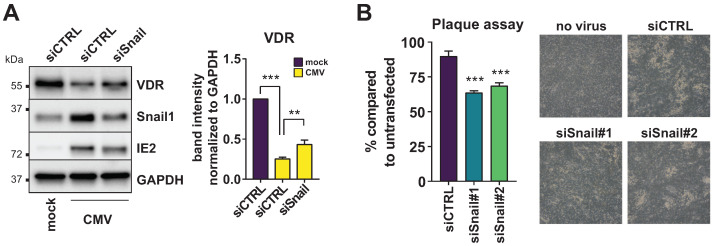
Snail ablation restores VDR expression. (**A**) VDR and Snail protein expression was determined in lysates of HFF transfected with a non-targeting control siRNA or Snail1 specific siRNA and harvested at 24 h p.i. Representative blot (left) and quantification of bands from multiple experiments relative to the GAPDH loading control (right). Asterisks show significant differences to the CMV siCTRL control sample as determined by ANOVA Dunnett’s multiple comparison post-hoc test. (**B**) Plaque assay showing the percentage of plaques in siRNA-transfected cells compared to an untransfected control. Asterisks show significant differences in two different Snail-targeting siRNAs to the CMV siCTRL control sample as determined by ANOVA Dunnett’s multiple comparison post-hoc test. Light microscope pictures of representative areas are shown on the right.

Next, we asked whether Snail induction might have a direct effect on VDR expression in the context of HCMV infection. Therefore, we targeted Snail1 for knockdown using a specific siRNA. Indeed, Snail ablation could moderately increase VDR protein levels in HCMV-infected cells compared to the non-targeting control (Figure 5A), suggesting that Snail upregulation might be directly linked to VDR repression during HCMV infection. However, Snail knockdown could not completely revert VDR expression to mock levels, indicating that additional factors likely play a role. To further address the biological relevance of Snail1 for HCMV infection, we next performed plaque assays with transient knockdown of Snail1 using two different siRNA mixes. Indeed, Snail knockdown led to a moderate, but significantly reduced, plaque number compared to the control transfection (Figure 5B), indicating that Snail1 upregulation, while not essential, might be an important accessory factor for HCMV propagation.

### 3.5. The Vitamin D Inducible Peptide Cathelicidin Prevents HCMV Infection In Vitro

**Figure 6:**
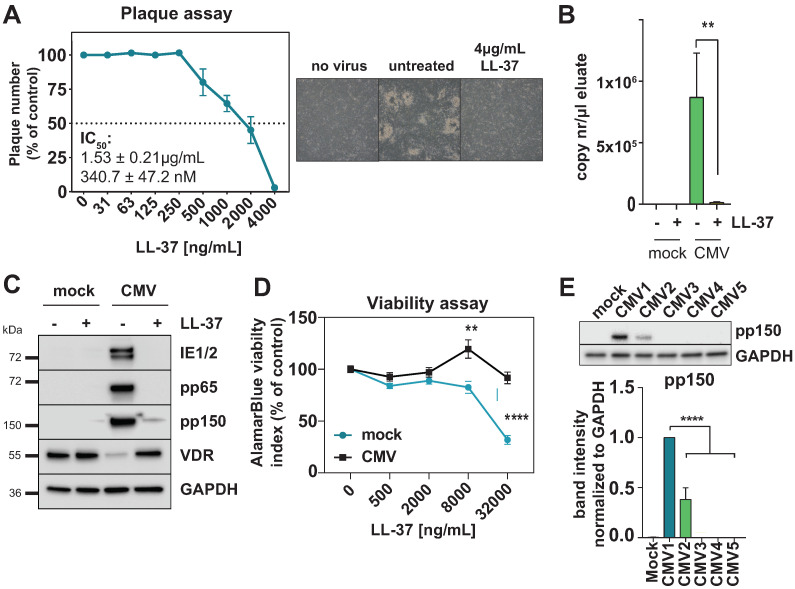
Cathelicidin inhibits HCMV replication. (**A**) Plaque reduction assay of HFF infected with HCMV AD169 pre-treated with increasing amounts of LL-37; percentage compared to DMSO control is shown (n = 2). Light microscope pictures of representative areas for the non-infected control, virus control and maximum LL-37 concentration are shown on the right. (**B**) Intracellular CMV DNA was detected in lysates from infected HFF at 72 h p.i. and 4 μg/mL LL-37 (where indicated) using primers specific for the UL83 (pp65) gene. Quantification of copy numbers per measured sample were calculated by interpolation of values against a 5-point standard curve of pcDNA-pp65 plasmid dilutions. (**C**) Western blot showing abundance of the indicated proteins in whole cell lysate (WCL) from mock-infected or HCMV-infected HFF at 96 h p.i with or without treatment with 4 μg/mL LL-37 peptide. Representative of three independent experiments. (**D**) AlamarBlue viability assay showing reduction in the viability index in mock- and CMV-infected cells treated with up to 32 μg/mL LL-37 compared to the respective untreated control. (**E**) Western blot (left) and quantification (right) of pp150 relative protein abundance in cell lysates harvested at 96 h p.i. at MOI 0.05. HFF cells were either left untreated (mock, CMV1) or LL-37-treatment conditions (4 μg/mL, CMV2 to CMV5). LL-37 was either added to the medium 2 h after inoculation (CMV2), or incubated for 1h with the cells prior to infection and then removed before adding the inoculum (CMV3). CMV4: LL-37 was incubated for 1h with the viral supernatant before inoculating the cells, or LL-37 was added to both the cells and viral supernatant (CMV5).

In a previous study, we showed that HCMV infection downregulates the VDR and calcitriol (the bioactive form of vitamin D) is not able to directly inhibit HCMV propagation in infected fibroblasts [13]. However, it is not known whether this mechanism is to the benefit of the virus. To assess the biological relevance of vitamin-D resistance, we thus tested the capacity of the vitamin-D-inducible antimicrobial peptide cathelicidin (LL-37) to directly inhibit human cytomegalovirus propagation. When added to the inoculum, cathelicidin significantly inhibited HCMV titers in a plaque reduction assay at an average IC50 of approximately 1.5 μg/mL (Figure 6A). Similarly, the intracellular accumulation of viral genome copies at 72 h post infection was almost completely abolished in cells treated with LL-37 (Figure 6B). HCMV immediate early (IE1/pp72 and IE2/pp86), early (pp65) and late proteins (pp150) were strongly repressed in lysates harvested at 96 h post infection (Figure 6C), suggesting a strong protective effect of LL-37. Importantly, LL-37 did not affect cell viability at the concentrations used in our assays (Figure 6D). Cathelicidins such as LL-37 consist of amphipathic helices and are thought to exert their antimicrobial functions mainly by directly targeting the viral envelope. However, it has also been suggested that cell-mediated mechanisms, such as the upregulation of interferon-stimulated genes, might contribute to its antiviral activities [28]. As seen in Figure 6E, CMV late antigen inhibition was strongest when the peptide could directly inactivate the viral inoculum or was added as a pre-treatment on the cells before the addition of virus. However, a 50% reduction was still observed if LL-37 was added to the cells after inoculation and removal of the virus, indicating that both virus and cell-targeting mechanisms might play a role in the CMV-inhibiting effect of LL-37. These results hint at the potential biological importance of VDR repression for HCMV viral propagation, and suggest a way to circumvent vitamin D resistance in infected cells by directly supplying vitamin-D-inducible antimicrobial peptides.

## 4. Discussion

In previous studies, we found that HCMV downregulates the vitamin-D receptor (VDR) in vitro, preventing potential direct antiviral effects of vitamin D and vitamin D analogs [13]. Additionally, we found that the VDR is reduced in peripheral blood of hematopoietic stem cell transplant (HSCT) patients undergoing active HCMV infection [14]. Mechanistically, it is not known how HCMV influences VDR expression and whether this is of any biological relevance. In this study, we established that the VDR is inhibited at the promoter level during HCMV infection (Figure 1). Treatment with the HDAC inhibitor TSA showed to reverse HCMV-induced VDR downregulation, and a ChIP assay further confirmed reduced H3K27 acetylation at the VDR promoter region during infection, pointing toward a mechanism of epigenetic repression. While the VDR is a well-studied transcription factor known for regulating more than 200 potential target genes by recruitment of epigenetic coactivators or corepressors [18], less is known about the transcriptional regulation of the VDR itself. Among the few known regulators of the VDR promoter are the transcriptional repressors Snail1 (Snail) and Snail2 (Slug) [20]. However, Snail2 has recently been shown to be downregulated by HCMV, among other EMT markers, resulting in a mesenchymal-to-epithelial (MET)-like phenotype [21]. We could reproduce this downregulation of Snail2 in infected HFF, but, surprisingly, its homolog Snail1 was strongly induced and inversely correlated with VDR expression throughout infection (Figure 2). Epigenetic repression of the VDR promoter via Snail has previously been described in the context of HIV-1 infection [29]. It thus might be a feature more common in viral infections and not entirely HCMV-specific, even though we previously excluded VDR downregulation at least in the context of adenovirus and influenza infection, arguing against a general mechanism [13]. The reported induction of a MET phenotype during HCMV infection [21] is in contrast to Snail1 upregulation as well as other studies that reported an EMT phenotype, enhanced cell proliferation, migration, and EMT marker upregulation in colorectal cancer-derived stem-cell-like cells [30]. Moreover, given the labile nature of both Snail1 mRNA and protein, it might be overlooked in transcriptomic and proteomic datasets, and detection might prove difficult in protein samples that are not stabilized by both protease and phosphatase inhibitors. Reciprocal expression of Snail1 and Snail2 has been reported in the context of some cancers, and might even be causally linked, since Snail1 is capable of directly repressing Snail2 [31,32].

In addition to VDR downregulation, we also found that its heterodimerization partner RXRα is reduced at late time points of infection (Figure 3E). The nuclear receptor RXRα does not only exert its function in RXR/VDR heterodimers, but can pair up with a variety of other nuclear receptors such as the retinoid acid receptor alpha (RARα) or the thyroid hormone receptor (TR-β) [33,34]. CMV might thus control the expression of multiple steroid hormones, also in light of a recent study that describes a role in progesterone inhibition during pregnancy using murine cytomegalovirus infection models [35].

Next, we found that transcription of immediate early genes was necessary for HCMV-induced VDR downregulation, and IE2 overexpression in the absence of infection sufficed to increase levels of Snail1 and moderately reduce VDR in a cell line (Figure 3). IE2 is known to interact with chromatin, and it has both repressive autoregulatory and transactivating functions, which are crucial for the activation of HCMV early genes [36,37]. It will thus be interesting to further investigate whether IE2 is directly involved in Snail1 activation and VDR-promoter repression. It will also be important to further put these findings into context, e.g., given the roles of VDR and Snail in mechanisms of placental differentiation and pregnancies with fetal growth restriction [38,39,40]. In theory, infection with HCMV might throw VDR and Snail expression in the placental trophoblast progenitor cells of pregnant mothers out of balance, resulting in complications for the fetus. To further investigate the biological relevance of VDR and Snail regulation during HCMV infection, it would thus be of high interest to investigate them in the context of congenital HCMV infection.

Snail1 proteins are inherently unstable and targeted for degradation mostly via phosphorylation and subsequent ubiquitination, with a very short half life [25,26]. Indeed, we found that Snail1 was partly phosphorylated in CMV-infected HFF lysates, and propose a mechanism of Snail1 stabilization via phosphorylation by the catalytic subunit of the DNA-dependent Protein Kinase (DNA-PK) (Figure 4B), which has been known to stabilize Snail via Ser100 phosphorylation during metastasis [27]. HCMV is known to heavily manipulate the DNA damage response, e.g., by mislocalizing checkpoint proteins [41] or disrupting the ataxia telangiectasia mutated protein damage response [42]. HCMV immediate early proteins can even directly stimulate the homology directed repair (HDR) pathway [43]. Snail upregulation might, thus, be a consequence of HCMV-induced DNA damage response pathway. Yet, we also found evidence that Snail1 and VDR expression might be directly linked, since siRNA-mediated knockdown of Snail1 led to partly restored levels of VDR expression in HCMV-infected cells (Figure 5A).

However, Snail ablation could not completely revert VDR expression to mock levels, perhaps due to the moderate knockdown efficiency achieved on the protein level, or because additional factors might play a role. It is noteworthy that Snail2 was also strongly regulated during HCMV infection (Figure 2), and as Snail2–Snail1 interactions have been described to cooperatively influence the VDR [44], it remains to be determined whether very early Snail2-mediated repression events during infection can trigger the epigenetic changes for VDR/Snail1 dysregulation via interconnected feedback loops.

To address the biological relevance of Snail1 for HCMV infection, we additionally knocked down Snail1 in plaque reduction assays and found significantly reduced plaque numbers compared to the control transfection (Figure 5B). Snail1 upregulation, while not essential, might, thus, be an important accessory factor for HCMV propagation.

Finally, we found that the vitamin-D-inducible antimicrobial peptide cathelicidin (LL-37) is a potent inhibitor of HCMV at an IC50 of 341 nM (1.5 μg/mL) (Figure 6). This is relatively low in comparison to the reported IC50 of LL-37 of 15 μM against HIV-1 [45] or 2 μM against HPV16 [46], suggesting a pronouncedly high susceptibility of HCMV to LL 37 in vitro. LL-37 is structured as an amphipathic helix with both a hydrophobic and a polar face, a feature that allows cathelicidins to interact with membranes and exert their antimicrobial activity by destroying them [47], and, thus, likely acting as a viral entry inhibitor. However, cathelicidins are not only able to target microbial membranes directly, but also stimulate several Toll-like receptors to raise alarm in surrounding cells [48,49]. Interestingly, LL-37 also plays an important role in the vitamin-D-induced autophagy of monocytes and macrophages [50]. On the other hand, calcitriol supplementation was shown to induce the HCMV lytic cycle in monocytes in vitro [51], likely associated with the ability of vitamin D to induce macrophage differentiation. Once HCMV-infected monocytes differentiate into macrophages, the lytic cycle is triggered and IE gene expression is detectable [52]. Since our data suggest that IE proteins might be necessary to induce the loss of VDR, VDR downregulation might theoretically constitute an important protective effect against the activation of antimicrobial peptides during lytic activation. It is tempting to speculate that VDR repression is an important immunoevasion mechanism which benefits HCMV viral propagation, and, in consequence, suggest circumventing vitamin-D resistance in infected cells by directly supplying vitamin-D-inducible antimicrobial peptides. However, more research will be necessary to assess the biological relevance of HCMV-induced vitamin-D resistance beyond the use of laboratory HCMV infection models, as used in our study.

HCMV infection makes infected cells refractory to vitamin-D signaling by VDR downregulation, in vitro but also in the blood of hematopoietic stem-cell transplant patients [14], and hematopoietic cells are a major source of LL-37 production [5]. This also highlights the potential of antimicrobial peptides as an antiviral treatment regimen, especially with regard to a defective vitamin-D system.

In summary, our work provides important insights into virally induced vitamin-D resistance. We have found that vitamin-D-inducible peptides are highly active against CMV. Thus, VDR downregulation might represent an important viral defense mechanism that may be targeted therapeutically.

## Data Availability

The data are available within the manuscript or its Supplementary Files.

## Supplementary Materials

The following supporting information can be downloaded at: https://www.mdpi.com/article/10.3390/v14092004/s1, Table S1: List of primers; Table S2: List of siRNAs.
