# Supplementary material for: Human Cytomegalovirus Induces Vitamin-D Resistance In Vitro by Dysregulating the Transcriptional Repressor Snail
Source: Viruses. 2022 Sep 10;14(9):2004. doi: 10.3390/v14092004 (PMC9505537; doi:10.3390/v14092004)
Supplement: Supplementary file 1 [file viruses-14-02004-s001.zip › viruses-1903299-supplementary.pdf]

Supplementary Information

Table S1 List of primers

Targeted gene	Forward	Reverse
GAPDH	5'- AAGGTGAAGGTCGGAGTCAAC-3'	5'-GGGGTCATTGATGGCAACAAT-3'
PGK1	5'- AAGTGAAGCTCGGAAAGCTTCTAT-3'	5'- AGGGAAAAGATGCTTCTGGG-3'
RXRα	5'- TTCTCCACCCAGGTGAACTC-3'	5'- GAGCTGATGACCGAGAAAGG-3'
SDHA	5'- TGGGAACAAGAGGGCATCTG-3'	5'- CCACCACTGCATCAAATTCATG-3'
Snail1#1	5'- TTTACCTTCCAGCAGCCCTA-3'	5'- GGACAGAGTCCCAGATGAGC-3'
Snail1#2	5'- CGAACTGGACACACATACAGTG -3'	5'- AGATGAGCATTGGCAGCGAG-3'
Snail2	5'- AAGGTGAAGGTCGGAGTCAAC-3'	5'- CTGAGGATCTCTGGTTGTGGT -3'
VDR	5'- TCTCCAATCTGGATCTGAGTG-3'	5'- ACAGCTCTAGGGTCACAGAAG-3'
VDR-precursor	5'- TGACCCTGGAGACTTTGACC-3'	5'- GGGAGAGCCTGGGAGGAG-3'
VDR promoter	5'- CTGCAGCAGTAACAGGTTGG-3'	5'- GCTTCAGCCTGTGTTAATCG -3'
IE2(cDNA)	5'- TTTCCATGGGTCTTTTCTGC-3'	5'- TACACATGAGGGGGAGAAGG-3'
IE2(BamHI/XbaI)	5'- TATGCGGATCCATGGAGTCCTCTGCCAAGA-3'	5'- TGCTTATCTAGATTACTGAGACTTGTTCCTCAG-3'

Table S2 List of siRNAs

Targeted gene	
siCTRL	5'- AAUUCUCCGAACGUGUCACGU-3'
siSnail	#1 5'- CCACAGAAAUGGCCAUGGGAAGGCC-3'<br>#2 sc-38398 (mix Santa Cruz Biotechnology)
siIE1/2	#1 5'- GGAAGAAAGUGAACAGAGUUU -3'<br>#2 5'- GGAAGGAGGUUAACAGUCAUU -3'
siDNAPK	#1 5'- GAUCGCACCUUACUCUGUU-3'<br>#2 5'- CUUUAUGGUGGCCAUGGAG-3'
